# The British Standard for (European Conformity[CE] Marked) Anti‐D: Its rarely discussed but important role in quantitating anti‐D in patient plasma

**DOI:** 10.1111/tme.12649

**Published:** 2019-11-26

**Authors:** Bernard Fox, Jason Hockley, Lucy Studholme

**Affiliations:** ^1^ Division of Biotherapeutics National Institute for Biological Standards and Control Hertfordshire UK; ^2^ Division of Analytical and Biological Sciences National Institute for Biological Standards and Control Hertfordshire UK

Dear Sir,

The National Institute for Biological Standards and Control (NIBSC) has been issuing the British Standard for Anti‐D, used to calibrate the AutoAnalyser^1^ for quantitation of anti‐D in patient plasma, for some 40 years. Recently, whilst establishing the current 3rd British Standard (CE Marked) for anti‐D in plasma, we realised that, although there are passing references in the literature,^2^, ^3^, ^4^, ^5^ the origins and establishment of this standard have never been published. First, we would like to provide a historical overview of how the first working standard was originally established and, second, how it has evolved, by reporting on the collaborative study to establish the current 3rd British Standard (CE Marked) for anti‐D in plasma.

In October 1971, the Directors of the UK National Transfusion Centres decided that a national working standard for anti‐D, for use in automated assay techniques, was required for routine use in hospitals. The candidate standard was prepared from 23 L of pooled citrated plasma containing incomplete (IgG) anti‐D from donors in early, mid and late stages of immunisation. Plasma was recalcified, the clot removed and excess calcium absorbed on an ion‐exchange resin, followed by 0.45 μm filtration into three separate containers for sterile storage. Subsequently, the material from each of the three containers was distributed (0.5 mL) into glass ampoules and lyophilised to generate three batches (NIBSC filling codes: 72/229; 73/515; and 73/517) and stored at −20°C. Analyses showed that each ampoule contained 0.58% residual moisture and 0.14% oxygen.

The three batches of lyophilised material, described above, were distributed in a collaborative study with the aim to establish a British Working Standard. These candidates were assayed against the International Standard (IS; 64/19) for Anti‐D Incomplete Blood Typing Serum^6^ using groups O, R_1_R_1_ and R_2_r cells on the AutoAnalyser by five UK clinical laboratories. The results showed that there was no difference between the dose‐response curves of the three candidate batches of lyophilised material. Consequently, data for all three batches were pooled for each laboratory, and anti‐D potencies, relative to the IS, were determined by parallel‐line analysis. There were signs of differences with the slope of pooled candidate data, which tended to be steeper than that of the IS, although non‐parallelism (*P* <0 .01) was only found in 2 of 80 assays, no more than would be expected by chance. Deviations from linearity were observed in 8 of 80 assays with a very small error, which was overcome by reducing the weights of these assays in the potency calculations. The potency estimates varied between laboratories. Two laboratories obtained potencies of 14 IU/ampoule, and the other three laboratories estimated around 10 IU/ampoule. The overall mean potency from all laboratories was 11.54 IU/ampoule (95% confidence interval [CI]: 11.00‐12.11). The discrepancy between laboratories was presumed to be a result of some variable in the assay system, which was not apparent from the information provided by the participants. There was, at the time, an immediate practical necessity to assign a universally acceptable value to the active content of one batch of proposed standard, and for this purpose, an overall mean potency from all laboratories was deemed adequate. Following this study, the 1st British Standard for Anti‐D (Rh_0_) antibodies intended for use in the assay of plasma anti‐D levels by the AutoAnalyser was established in 1975 with an assigned potency of 11.5 IU/ampoule and was coded 72/229.

In 1988, when stocks of 72/229 were running low, it was proposed that 73/515 should replace 72/229 as it was prepared from the same plasma pool and was included in the original collaborative study described above. This time, four UK transfusion centres undertook to assay 73/515 against 72/229 using the AutoAnalyser, and the results of this study showed 73/515 to be indistinguishable from 72/229, with the potencies falling within the range of potencies obtained for the original collaborative study. To ensure continuity, in 1992, 73/515 was adopted as the 2nd British Standard for anti‐D antibodies with an assigned potency of 11.5 IU/ampoule.

In 2005, using the conformity assessment route, 73/515 was CE marked under Directive 98/79/EC on in vitro diagnostic medical devices and complied with the UK Guidelines for the Blood Transfusion Services.

Most recently, a collaborative study was carried out involving three UK transfusion centres experienced in AutoAnalyser methodology to assess the stability of anti‐D in lyophilised preparation 73/517 and its suitability to replace the CE‐marked standard (73/515). Each participant was provided with two ampoules of 73/515 (stored at −20°C since lyophilisation) and eight ampoules of 73/517 (two of each stored at −70°C, −20°C, +4°C and +20°C for 11 years prior to assay). They were required to reconstitute, on the day of assay, one ampoule each of 73/515 and 73/517 from each storage temperature. On each of the 2 days, the labs were required to perform two independent assays following NHSBT protocols using a fresh dilution series for each. Potencies for 73/517 assayed against the current standard (73/515) were centrally calculated, at NIBSC, by parallel‐line analysis using the European Directorate for the Quality of Medicines software, CombiStats v5.0, and are shown in Table 1a. All results were linear and parallel, demonstrating that the participating laboratories were highly competent, obtaining an unweighted geometric mean potency of 11.6 IU/ampoule (95% CI: 10.5‐12.8).

**Table 1:** Potency and stability results for the candidate 3rd British Standard for anti‐D

a) Potency results for 73/517 (candidate 3rd British Standard) assayed against 73/515 (2nd British Standard)				
Lab	Day	95% LCL	GM Potency for 73/517 (IU/ampoule)	95% UCL
1	1	10.9	12.0	13.1
1	2	11.3	12.5	13.9
2	1	10.8	11.3	11.8
2	2	10.5	13.0	16.5
3	1	9.7	10.0	10.4
3	2	10.5	11.0	11.5
		95% LCL	GM combined lab potency	95% UCL
		10.5	11.6	12.8

b) Combined lab potency results for 73/517 stability samples assayed against the −70°C reference sample				
Temp (°C)	Time (year)	95% LCL	GM potency (IU/ampoule)	95% UCL
−20	11	11.2	11.5	11.9
+4	11	11.3	11.6	12.0
+20	11	7.9	10.4	13.6

GM, geometric mean; LCL, lower confidence limit; UCL, upper confidence limit.

In addition, from the (loss of) potency in ampoules stored at the elevated temperatures compared to the potency of ampoules stored at −70°C, we can estimate the long‐term stability^7^ at −20°C, which is the storage temperature of our stock. This is an indirect method used to determine the rate of loss based on the relationship between reaction rates and temperature given by an Arrhenius equation and where a first‐order reaction rate is assumed.^8^ After 11 years of storage at elevated temperatures, we were able to show that 73/517 would continue to be sufficiently stable (<0.01% loss per annum at −20°C) for at least another 10 years. Table 1b shows the potency results for 73/517 stability samples.

The measurement of anti‐D levels in plasma are carried out for determining and monitoring antibody levels during and after pregnancy; decisions for clinical treatment are made on the basis of assay results,^3^, ^9^ and hence, there is a requirement for accurate standardisation. In the 1970s, interlaboratory variability of up to ±100% of the mean was not uncommon when using non‐standardised methods.^5^ Since the introduction of a common standard and the use of standardised protocols, there has been a significant reduction in interlaboratory variability to around 20%.^10^ In another recent study,^4^ the three laboratories performing AutoAnalyser quantitation for therapeutic hyperimmune anti‐D had interlaboratory variability ranging from 6% to 14% depending on the test sample. To maintain continuity with the 2nd British Standard (73/515), 73/517 was established as the 3rd British Standard for Anti‐D with an assigned potency of 11.5 IU/ampoule, complying with the UK Guidelines for the Blood Transfusion Services, and continues to be CE marked. 73/517 is available from the NIBSC.

## CONFLICT OF INTEREST

The authors have no competing interests.
